# Multiparametric functional magnetic resonance imaging for evaluation of hepatic warm ischemia-reperfusion injury in a rabbit model

**DOI:** 10.1186/s12876-017-0720-8

**Published:** 2017-12-15

**Authors:** Qian Ji, Zhi Qiang Chu, Tao Ren, Shi Chao Xu, Long Jiang Zhang, Wen Shen, Guang Ming Lu

**Affiliations:** 1 Department of Medical Imaging, Jinling Hospital, Medical School of Nanjing University, 305 Zhongshan East Road, Xuanwu District, Nanjing, Jiangsu China; 2 Department of Radiology, Tianjin First Central Hospital, 24 Fukang Road, Nankai District, Tianjin, China; 3 Department of Transplantation, Tianjin First Central Hospital, 24 Fukang Road, Nankai District, Tianjin, China

**Keywords:** Donation after cardiac death (DCD), Hepatic warm ischemia-reperfusion injury (WIRI), Diffusion weighted imaging (DWI), Intravoxel incoherent motion (IVIM), Diffusion tensor imaging (DTI), Blood oxygen level–dependent (BOLD)

## Abstract

**Background:**

To assess the feasibility of noninvasive and quantitative evaluation of hepatic pathophysiological changes in rabbit hepatic warm ischemia-reperfusion injury (WIRI) models by using intravoxel incoherent motion (IVIM), diffusion tensor imaging (DTI) and blood oxygen level dependent (BOLD) MRI.

**Methods:**

Twenty rabbits were randomly divided into hepatic WIRI model group and sham-operation group (*n* = 10 for each group). Hepatic WIRI was induced in rabbit by occluding hepatic inflow for 30 min and reperfusion for 6 h. The control group only underwent laparotomy and liver ligament dissection. IVIM with 11 b values (0 to 800 s/mm^2^), DTI with 2 b values (0 and 500 s/mm^2^) on 12 diffusion directions, and BOLD MRI with 9 TE (2.57 to 24.25 ms) were performed at 3 T clinical MR scanner. Rabbits were sacrificed for biochemical and histopathological analysis after MR scanning. All of functional MR, biochemical and histopathological parameters were analyzed by independent sample *t* test, Mann-Whitney U test, Pearson and Spearman correlation methods.

**Results:**

All of MR parameters showed moderate to excellent interobserver reproducibility. True diffusion (Dslow), pseudodiffusion (Dfast), perfusion fraction (PF), and mean diffusitivity (MD) were lower in WIRI models than in control rabbits (*P* < 0.01), R2* was higher in WIRI models than in control rabbits (*P* < 0.001), while fractional anisotropy (FA) showed no statistical difference. There were significant differences in I score and all of biochemical parameters between the two groups (*P* < 0.01). Functional MR parameters corresponded well with all of biochemical parameters and some of histopathological parameters (*P* < 0.05). Histopathological analysis showed the structure and morphology of hepatic lobule was normal and clear in control rabbits, while diffuse hepatocyte swelling, central vein and sinusoids congestion, and inflammatory cell infiltration in WIRI models.

**Conclusions:**

IVIM, DTI, and BOLD MRI are noninvasive and useful techniques for assessing the microenvironment changes of hepatic WIRI in rabbit models.

## Background

Hepatic ischemia–reperfusion injury (IRI) is the most common cause of liver damage during liver transplantation [1]. Donation after cardiac death (DCD) has expanded the donor pool for liver transplantation, especially in Asian countries [2–4]. However, compared with donation after brain death and living donation, DCD liver grafts are associated with significantly worse outcome [5–9]. The hepatic IRI of DCD liver grafts is attributed to blood supply returns to the tissue after a period of ischemia during graft harvest (also called warm ischemia), graft storage (cold ischemia) and graft implantation (reperfusion). The extent of injury during cold ischemia and reperfusion depends on the duration during the warm ischemia. Reperfusion of ischemic tissue is essential for survival, it also initiates oxidative damage, cell death and aberrant immune responses through the generation of mitochondrial reactive oxygen species (ROS) [10]. Therefore, Hepatic warm ischemia–reperfusion injury (WIRI) causes sever ischemia and anoxia, oxidation/antioxidation imbalance, and excessive inflammatory response [11], which result in liver microcirculatory disorders and histological damage.

Although liver biopsy is considered the standard technique for diagnosis of liver damage, it is impossible to be a routine and dynamic monitoring method, because it is an invasive method with possible complications and sampling error [12, 13]. Instead, MR imaging has played an important role in this setting [14–18]. Intravoxel incoherent motion (IVIM) MRI and diffusion tensor imaging (DTI) are advanced techniques based on diffusion-weighted imaging (DWI). IVIM allows for separation perfusion (or microcirculation) related diffusion from pure molecular diffusion, by analyzing the signal decay of multi-b-value DWI [19, 20]. DTI can reveal microstructural characteristics of biological tissue, which can detect the degree of diffusion in multiple dimensions by using at least six or more gradient directions [21, 22]. Blood oxygen level–dependent (BOLD) MRI is a MRI technique that using deoxyhemoglobin as an endogenous contrast to reflect alterations in blood oxygenation, blood flow, and blood volume [23]. To our knowledge, a limited number of studies have investigated the feasibility of IVIM, DTI and BOLD MRI to characterize liver fibrosis [23, 24], liver cirrhosis [25], and hepatic tumors [26–28], while only a few reports assessed the role of a single technique above mentioned in the hepatic WIRI [18, 29].

Therefore, we attempt to determine the feasibility of noninvasive evaluation and characterization of hepatic diffusion, perfusion and oxygenation changes of hepatic WIRI in a rabbit model by comprehensively using IVIM, DTI and BOLD MRI.

## Methods

### Animal model

This study was approved by the local Animal Experimentation Ethics Committee and performed in accordance with institutional guidelines (No: 2017N030KY). Twenty adult New Zealand White rabbits weighing 2.5-3.0 kg were randomly divided into two groups: hepatic WIRI model group (*n* = 10) and sham-operation control group (n = 10). All of rabbits were anesthetized with intravenous injection of 5% amobarbital (Sigma-Aldrich Chemical, St. Louis, MO, USA) at a dose of 1 mL/kg. Hepatic WIRI was induced by temporarily clamping the hepatic artery and portal vein for 30 min and reperfusion for 6 h, followed by MRI studies. The control group only underwent laparotomy and liver ligament dissection. All experimental procedures were performed by a well-experienced surgeon (with 15 years of experience in general surgery).

### MR imaging

MR data acquisition was performed under 6-channel phased array body coil of 3 T clinical system (MAGNETOM Trio Tim, Siemens Healthcare, Erlangen, Germany). All rabbits were anesthetized with intramuscular injection xylazine hydrochloride (Huamu Animal, Jilin, China) at a dose of 0.2 mL/kg, and then positioned supinely with a belt to reduce the respiratory motion.

Axial IVIM images were acquired using a free-breathing single-shot echo-planar imaging (ss-EPI) prototype sequence with the following parameters: TR/TE of 1000/57.2 ms, EPI factor of 96, voxel size of 1.9 × 1.9 × 4.0 mm^3^, field of view (FOV) of 180 × 180 mm^2^, slice thickness of 4 mm, matrix of 96 × 96, with 3 measurements averaged and an acquisition time of 2 min. DW gradients (i.e. 11 b values of 0, 20, 40, 60, 80, 100, 150, 200, 400, 600, and 800 s/mm^2^) were applied in three orthogonal directions and were subsequently averaged.

Axial DTI images were acquired using a free-breathing ss-EPI prototype sequence with the following parameters: TR/TE of 3800/86 ms, EPI factor of 88, voxel size of 1.4 × 1.4 × 4.0 mm^3^, FOV of 180 × 180 mm^2^, slice thickness of 4 mm, matrix of 128 × 128, with two b values of 0 and 500 s/mm^2^ on 12 diffusion directions, and an acquisition time of 309 s.

All of animals were exposed to room air (78% N_2_, 20% O_2_) to maintain normoxia state. Liver BOLD imaging was performed using a multi-echo gradient echo pulse sequence with free-breathing examination to acquire axial T2*-weighted images. Other imaging parameters included: TR of 75 ms, TE of 2.57-24.25 ms (9 echoes), flip angle of 30°, voxel size of 2.0 × 1.6 × 4.0 mm^3^, FOV of 300 × 225 mm^2^, matrix of 192 × 154, slice thickness of 4 mm, number of slices 18, and a total examination time of 76 s.

### Image analysis

Image analysis was performed with prototype software supplied by the manufacturer (Siemens Healthcare, Erlangen, Germany). Three IVIM-derived parameters (Dslow: the true diffusion as reflected by pure molecular diffusion, Dfast: the pseudodiffusion coefficient related to perfusion, and PF: the perfusion fraction which represents a fractional volume of microcirculation within a voxel) were calculated by a full bi-exponential fitting of the MR signal intensity images of all b values on a pixel-by-pixel basis, using the equation as previously described [30].

For quantitative analysis of DTI, the mean diffusitivity (MD) and fractional anisotropy (FA) maps were generated automatically as previously described from all diffusion-weighted images on the postprocessing workstation. The range of FA value is from 0 to 1 [26].

BOLD-derived T2* (ms) and R2* (1/T2*, sec^−1^) were calculated by fitting a monoexponential, voxel by voxel, to the signal intensity-echo time curve [23, 31]. Color-coded parametric images of R2* were obtained using commercial software (Image J, NIH, Bethesda, MD, USA).

All regions of interest (ROIs) were manually positioned by two radiologists (with 10 and 5 years of experience in abdominal MR imaging) on images with b value of 0 s/mm^2^, and then were copied to the corresponding Dslow, Dfast, PF, MD, FA, and T2* maps. Six circular ROIs were manually drawn on 3 central slices of the liver to avoid the inclusion of large intrahepatic vessels/bile ducts and the margin. The mean size of all ROIs in the liver is 12 pixels ±2. The mean value of the six ROIs was used for the final analysis.

### Biochemical and Histopathological analysis

After MR scanning, 4 ml blood was collected from the auricular vein of each animal. The blood was centrifuged to obtain serum. Serum liver function parameters, including alanine transaminase (ALT), aspartate transaminase (AST), and lactate dehydrogenase (LDH) were measured using automatic biochemistry analyzer (HITACHI TAB-40F2, Japan) with standard procedures. The rabbits were then sacrificed by an overdose of 5% amobarbital by intravenous injection, and 1 g liver tissue were collected for each animal. The oxidant/antioxidant, and inflammatory parameters, including malondialdehyde (MDA), superoxide dismutase (SOD), and myeloperoxidase (MPO) of liver tissue were measured using spectrophotometry method (Jiancheng Bio, Nanjing, China).

Liver specimens were fixed in 4% phosphate-buffered formaldehyde and embedded in paraffin. After that, hematoxylin and eosin (H&E) staining was performed on each section for histological analysis. All HE-stained tissue sections were examined microscopically under an optical microscope (Olympus BX50, Japan). The stages of hepatic inflammation/necrosis (I score) and liver fibrosis (F score) were scored by one histopathologist (with 16 years of liver pathology) using the METAVIR classification system [32] and blinded to the results of the group and MR.

### Statistical analysis

Interobserver reproducibility was assessed by measuring intraclass correlation coefficients (ICC) [33]. Statistical analysis was performed using independent sample *t* test, and Mann-Whitney U test for two group comparisons of the functional MR parameters and biochemical parameters. The Pearson and Spearman correlation coefficient was calculated to assess the correlations between functional MR parameters and biochemical parameters. The data were analyzed using SPSS 17.0 software (SPSS, Chicago, IL, USA), *P* < 0.05 was considered statistically significant.

## Results

### Interobserver agreement of functional MR parameters

For IVIM, Dslow and PF values had excellent interobserver agreement with ICC of 0.960 (0.839-0.990), and 0.926 (0.701-0.982), respectively, and Dfast values showed moderate agreement with ICC of 0.663 (0.355-0.916). For DTI, MD and FA values showed excellent interobserver agreement with ICC of 0.890 (0.722-0.956), and 0.823 (0.554-0.930), respectively. For BOLD, R2* values showed excellent interobserver agreement with ICC of 0.870 (0.671-0.948).

### Comparison of functional MR parameters between two groups

**Fig. 1:**
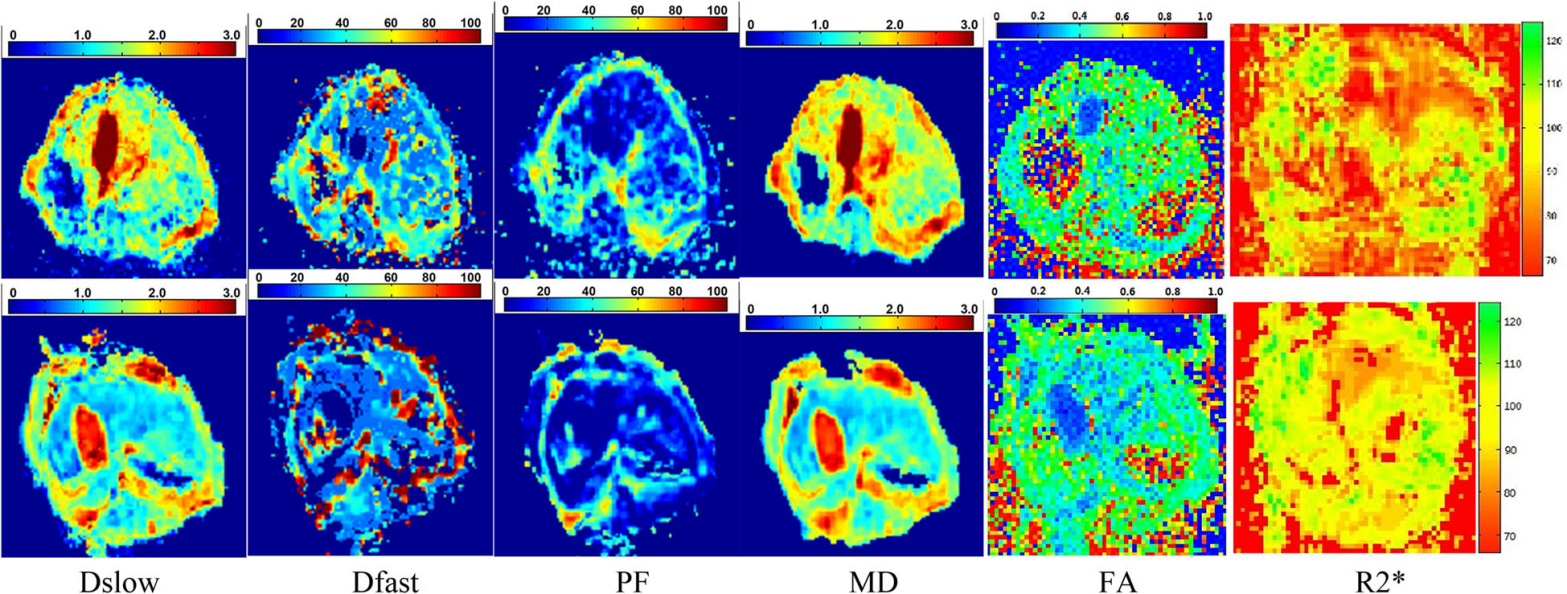
Typical IVIM, DTI and BOLD MRI parametric maps of a control rabbit (upper row) and a hepatic WIRI model rabbit (lower row). Dslow, Dfast, PF, and MD maps show lower signals, while R2* show higher signals in the rabbit with hepatic WIRI (lower row) than in the rabbit with normal liver (upper row)

Dslow, Dfast, PF, and MD of model group were significantly lower than control group (*P* < 0.01), while R2* of model group was significantly higher than control group (*P* < 0.001). FA changes between two groups was not statistically significant (*P* > 0.05) (Table 1). The corresponding typical examples of functional MR parametric maps were shown in Fig. 1. Compared with control group, Dfast and PF in model group decreased 25.01 ± 16.69%, and 34.45 ± 9.63%, respectively, while R2* increased 36.38 ± 18.31%.

**Table 1:** Functional MR measurements between two groups

MRI Parameters	Control group	Model group	*t* value	*P* value
Dslow (×10^−3^ mm^2^/s)	1.29 ± 0.14	1.04 ± 0.21	−2.721	0.007
Dfast (×10^−3^ mm^2^/s)	32.33 ± 8.87	22.99 ± 1.59	3.276	0.009
PF (%)	30.44 ± 2.80	19.97 ± 3.39	7.533	0.000
MD (×10^−3^ mm^2^/s)	1.76 ± 0.20	1.46 ± 0.14	3.952	0.001
FA	0.36 ± 0.03	0.38 ± 0.04	−1.362	0.190
R2* (sec^−1^)	88.89 ± 12.77	119.34 ± 7.53	−6.496	0.000

*Dslow* true diffusion or pure molecular diffusion, *Dfast* pseudodiffusion or perfusion related diffusion, *PF* perfusion fraction, *MD* mean diffusitivity, *FA* fractional anisotropy

### Laboratory findings and correlation analysis

There were significant differences in ALT, AST, LDH, MDA, MPO, and SOD between the two groups (*P* < 0.01) (Table 2). ALT, AST, LDH, MDA, and MPO of model group were significantly higher than control group (*P* < 0.01), while SOD of model group was significantly lower than control group (*P* < 0.001). The correlation analysis showed that there were significant negative correlations between Dslow, Dfast, PF, MD and liver function, oxidant, inflammatory parameters (ALT, AST, LDH, MDA, MPO) (all *P* < 0.05), and positive correlations between Dslow, Dfast, PF, MD and antioxidant parameter (SOD) (all *P* < 0.05). Converse results were seen in the correlations between R2* and biochemical parameters (all *P* < 0.05). There were no significant correlations between FA and biochemical paratemers (all *P* > 0.05). These results are summarized in Table 3.

**Table 2:** Summary of biochemical and histopathological parameters between two groups

Group	ALT (U/L)	AST (U/L)	LDH (U/L)	MDA (nmol/ml)	MPO (U/G)	SOD (U/ml)	I score	F score
Control group	34.50(24.75,37.00)	44.70 ± 31.23	314.20 ± 285.60	2.06 ± 0.34	14.22 ± 3.87	183.20 ± 20.89	0	0
Model group	165.00(65.00,247.75)	348.00(297.75,708.25)	1117.00(789.25,2024.75)	3.44 ± 0.43	19.29 ± 2.84	121.78 ± 11.92	1(0,1)	0.5(0,1)
*t*/Z value	−3.484	−3.628	−3.326	−7.950	−4.725	−3.781	−3.162	−2.517
P value	0.000	0.000	0.001	0.000	0.000	0.000	0.007	0.063

*ALT* alanine transaminase, *AST* aspartate transaminase, *LDH* lactate dehydrogenase, *MDA* malondialdehyde, *MPO* myeloperoxidase, *SOD* superoxide dismutase

**Table 3:** Summary of correlations between functional MR parameters and biochemical, histopathological parameters

Parameters	ALT(U/L)	AST(U/L)	LDH(U/L)	MDA(nmol/ml)	MPO(U/G)	SOD(U/ml)	I score	F score
Dslow(×10^−3^ mm^2^/s)	−0.568 ^a^	−0.513	−0.707	−0.611	−0.514	0.549	−0.609	−0.441
	0.009 ^b^	0.021	0.000	0.004	0.021	0.012	0.004	0.052
Dfast(×10^−3^ mm^2^/s)	−0.711	−0.600	−0.586	−0.749	−0.676	0.630	−0.552	−0.451
	0.000	0.005	0.007	0.000	0.001	0.003	0.012	0.045
PF(%)	−0.670	−0.662	−0.726	−0.835	−0.760	0.772	−0.715	−0.471
	0.001	0.001	0.000	0.000	0.000	0.000	0.000	0.036
MD(×10^−3^ mm^2^/s)	−0.454	−0.542	−0.693	−0.616	−0.460	0.568	−0.831	−0.631
	0.044	0.014	0.001	0.004	0.041	0.009	0.000	0.003
FA	0.024	0.313	0.175	0.316	−0.073	−0.414	0.139	0.336
	0.919	0.179	0.462	0.175	0.760	0.070	0.559	0.147
R2*(sec^−1^)	0.756	0.802	0.680	0.760	0.485	−0.792	0.445	0.390
	0.000	0.000	0.001	0.000	0.030	0.000	0.049	0.089

*ALT* alanine transaminase, *AST* aspartate transaminase, *LDH* lactate dehydrogenase, *MDA* malondialdehyde, *MPO* myeloperoxidase, *SOD* superoxide dismutase, *Dslow* true diffusion or pure molecular diffusion, *Dfast* pseudodiffusion or perfusion related diffusion, *PF* perfusion fraction, *MD* mean diffusitivity, *FA* f ractional anisotropy

^a^upper row: *r* value is obtained from Spearman correlation analysis between two parameters

^b^lower row: *P* value is obtained from Spearman correlation analysis between two parameters

### Histopathological analysis

**Fig. 2:**
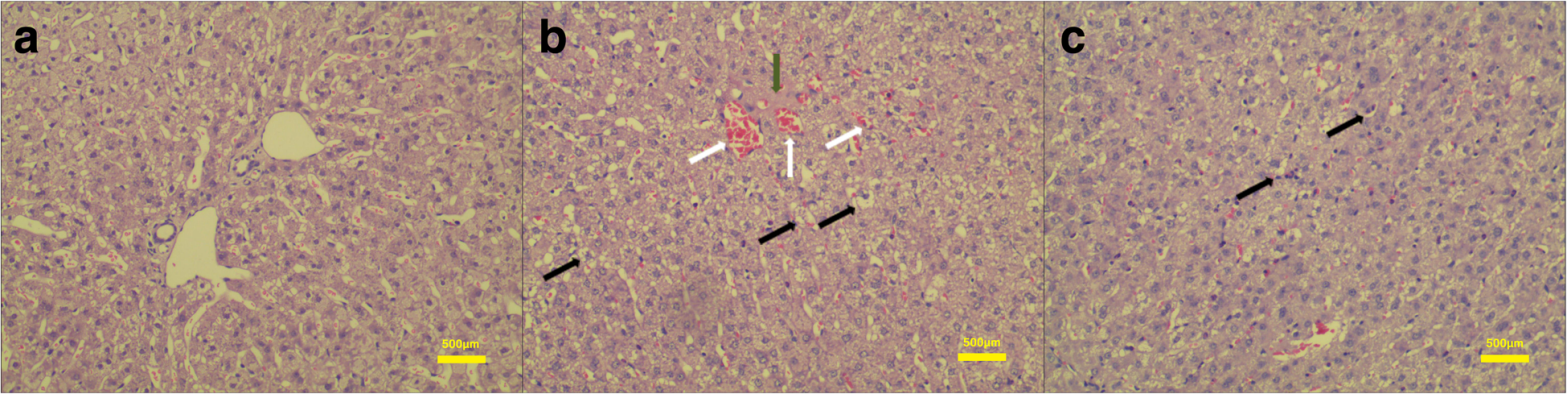
Hematoxylin-eosin (HE) staining images of liver. **a**. a control rabbit (magnification, ×100), the structure and morphology of hepatic lobule was normal and clear, there were no hepatocyte edema, sinusoids congestion and inflammatory cell infiltration. **b** and **c**. different regions of a hepatic WIRI rabbit model (magnification, ×100). **b** with regions of hepatocyte swelling indicated by black arrows, central vein and sinusoids congestion indicated by white arrows, and mild proliferation of fibrous tissue indicated by green arrow. **c** with regions of inflammatory cell infiltration indicated by black arrows

HE staining revealed that in the control group, the structure and morphology of hepatic lobule was normal and clear, there were no hepatocyte edema, sinusoids dilatation and inflammatory cell infiltration. Conversely, all hepatic WIRI rabbits had evidence of hepatocyte edema, inflammatory infiltration, central vein and sinusoids congestion, and mild proliferation of fibrous tissue (Fig. 2).

There were significant differences in I score (*P* < 0.01), while no significant differences in F score between two groups (*P* > 0.05) (Table 2). The correlation analysis showed that there were significant correlations between I score, F score and some of the functional MR parameters (*P* < 0.05) (Table 3).

## Discussion

In our preliminary sudy, we have proved the feasibility and reproducibility of IVIM, DTI and BOLD MRI as noninvasive alternatives to characterize the microenvironment changes of hepatic WIRI.

There have been no convincing data on the changes in liver IVIM and DTI study prior to this report [18, 29]. Our study demonstrated that there were significant differences in IVIM-derived Dslow, Dfast, PF values, DTI-derived MD values, and BOLD-derived R2* values between the two groups, which indicated that IVIM, DTI and BOLD MRI were sensitive to hepatic WIRI. Meanwhile, all of these parameters corresponded well with liver function (ALT, AST, and LDH), oxidant/antioxidant (MDA and SOD), and inflammatory parameters (MPO), which indicated that IVIM, DTI and BOLD MRI can effectively characterize the hepatic cellular injury and microscopic alteration of hepatic WIRI.

Histopathologically, in the hepatic WIRI model, hepatocyte swelling and inflammatory cell infiltration restrict the water molecular diffusion, leading to the decrease of hepatic diffusion (Dslow and MD values). In the present study, we first found that there were significant differences in I score between model group and control group, which confirmed the hepatocyte death, swelling and inflammatory cell infiltration in hepatic WIRI tissue. On the other hand, occlusion of hepatic artery and portal vein resulted in hepatic ischemia and anoxia, central vein and sinusoids congestion, and presence of “no-reflow” phenomenon after reperfusion [34], which causes reduced hepatic perfusion and oxygenation, that is, decrease of Dfast, PF values, and the increase of R2* value.

There is only one study in the literature on the use of DTI for hepatic IRI, in which a 7 T scanner was used [18]. They reported that FA 2 h after 30-min total hepatic IRI was significantly higher than that before and 1 day after IRI. In contrast to this study, although the FA showed a trend toward higher values in hepatic WIRI group, there was no statistical difference in FA between two groups in our study. So further studies are required to clarify where or not species difference is also one of the factors responsible for this variation in FA value in addition to differences in imaging protocol.

BOLD MRI can monitor the oxygenation state and blood perfusion of tissue [35]. The interpretation of BOLD MRI may be difficult in some situations, because both decreased blood flow and low oxygenation level can result in increased R2* value. For the first time, we attempt to evaluate the feasibility of characterization of hepatic WIRI in a rabbit model by using BOLD MRI. To avoid the documented effects of increasing glucose levels on blood flow [36] and R2* [37] in the liver, all rabbits were imaged in fasting conditions in our study. We found that when compared with control group, Dfast decreased 25.01 ± 16.69% in model group, while R2* increased 36.38 ± 18.31%. Therefore, combined application of BOLD MRI and IVIM technique, which can evaluate tissue perfusion without using of contrast agent, provide a better understanding both tissue oxygenation states and tissue perfusion, and in-depth assessment of hepatic WIRI pathophysiological changes.

Our study has certain limitations. First, we studied a small number of rabbits. However, this was an exploratory pilot study and herein we reported our preliminary findings. Second, hepatic WIRI is a dynamic process which is intrinsically time-dependent, while the model in this study was characterized only at a specific time frame. A longitudinal follow-up study may reveal the changes of disease evolution, which should be investigated in further studies. Third, we assessed only steady-state normoxia BOLD imaging rather than using extrinsic challenges, such as inhaled carbogen gas or 100% oxygen [23, 28, 38]. However, the use of extrinsic gas stimulation is technically difficult and time consuming. Our results demonstrated a statistically significant difference, which mean room air was sufficient to draw conclusions.

## Conclusions

IVIM, DTI, and BOLD MR imaging are useful and noninvasive techniques for evaluating in vivo hepatic microenvironment changes in a rabbit hepatic WIRI model.

## Abbreviations

ADC: Apparent diffusion coefficient
ALT: Slanine transaminase
AST: Aspartate transaminase
BOLD: Blood oxygen level–dependent
DCD: Donation after cardiac death
Dfast: Pseudodiffusion or perfusion related diffusion
Dslow: True diffusion or pure molecular diffusion
DTI: Diffusion tensor imaging
DWI: Diffusion weighted imaging
FA: Fractional anisotropy
HE: Hematoxylin and eosin
ICC: Intraclass correlation coefficients
IVIM: Intravoxel incoherent motion
LDH: Lactate dehydrogenase
MD: Mean diffusitivity
MDA: Malondialdehyde
MPO: Myeloperoxidase
PF: Perfusion fraction
ROI: Regions of interest
SOD: Superoxide dismutase
TNF-α: Tumor necrosis factor-α
WIRI: Hepatic warm ischemia-reperfusion injury
